# Unexpected Acceptance? Patients with Social Anxiety Disorder Manifest their Social Expectancy in ERPs During Social Feedback Processing

**DOI:** 10.3389/fpsyg.2015.01745

**Published:** 2015-11-16

**Authors:** Jianqin Cao, Ruolei Gu, Xuejing Bi, Xiangru Zhu, Haiyan Wu

**Affiliations:** ^1^Department of Nursing, Harbin Medical UniversityDaqing, China; ^2^Key Laboratory of Behavioral Science, Institute of Psychology, Chinese Academy of SciencesBeijing, China; ^3^Institute of Cognition and Behavior, Henan UniversityKaifeng, China

**Keywords:** feedback-related negativity (FRN), P2, outcome evaluation, social anxiety disorder, social rejection

## Abstract

Previous studies on social anxiety have demonstrated negative-expectancy bias in social contexts. In this study, we used a paradigm that employed self-relevant positive or negative social feedback, in order to test whether this negative expectancy manifests in event-related potentials (ERPs) during social evaluation among socially anxious individuals. Behavioral data revealed that individuals with social anxiety disorder (SAD) showed more negative expectancy of peer acceptance both in the experiment and in daily life than did the healthy control participants. Regarding ERP results, we found a overally larger P2 for positive social feedback and also a group main effect, such that the P2 was smaller in SAD group. SAD participants demonstrated a larger feedback-related negativity (FRN) to positive feedback than to negative feedback. In addition, SAD participants showed a more positive ΔFRN (ΔFRN = negative – positive). Furthermore, acceptance expectancy in daily life correlated negatively with ΔFRN amplitude, while the Interaction Anxiousness Scale (IAS) score correlated positively with the ΔFRN amplitude. Finally, the acceptance expectancy in daily life fully mediated the relationship between the IAS and ΔFRN. These results indicated that both groups could differentiate between positive and negative social feedback in the early stage of social feedback processing (reflected on the P2). However, the SAD group exhibited a larger FRN to positive social feedback than to negative social feedback, demonstrating their dysfunction in the late stage of social feedback processing. In our opinion, such dysfunction is due to their greater negative social feedback expectancy.

## Introduction

Social anxiety disorder (SAD) is characterized by fear of negative evaluation from others in social contexts according to the Diagnostic and Statistical Manual of Mental Disorders IV (American Psychiatric Association, 2000). Such intense fear of social evaluation is associated with a negative cognitive bias (i.e., negative-expectancy bias), which in turn impairs social ability in daily life. The cognitive-behavioral model of social anxiety proposes that socially anxious individuals assume that other people are inherently critical (Rapee and Heimberg, 1997) and have a negative-expectancy bias, i.e., severely socially anxious individuals hold a generalized belief that other people tend to evaluate them more negatively and underestimate their social performance (Leary et al., 1988; Alden and Wallace, 1995; Spence et al., 1999). For instance, socially anxious participants rated interviewers as having more negative opinions about them (Pozo et al., 1991). The existence of such a negative-expectancy bias was also confirmed in a recent study, which indicated that highly socially anxious individuals showed lower expectancy of positive social feedback in a two-visit task (Caouette et al., 2015). In a word, converging evidences have suggested that socially anxious individuals and patients with SAD demonstrate a negative bias in their expectancy for, and interpretation of, social evaluation (Amin et al., 1998; Messenger et al., 2004; Franklin et al., 2005; Creswell et al., 2014).

According to the cognitive-behavioral models of social anxiety, the relationship between cognitive processes (negative beliefs in social contexts) and social behaviors (perpetuated avoidance and withdrawal) is the core mechanism that comprises and maintains social anxiety (Heimberg et al., 2010). The negative-expectancy bias is one of the key cognitive aspects of SAD and plays a important role in the core mechanism of social anxiety. First, it leads to withdrawal or avoidance behavior among socially anxious individuals (Bogels and Mansell, 2004; Stirling et al., 2006), which results in poor social performance. Furthermore, negative evaluation of their performance may further lower their level of self-esteem and reinforce their negative belief (Leary, 1990; Stopa and Clark, 2000; de Jong, 2002; Amir et al., 2005; Laposa et al., 2010).

However, to our knowledge, there has been no direct evidence of the neural correlates of social feedback processing in SAD, which could demonstrate the relationship between social evaluation expectancy and outcome evaluation. The primary aim of the present study, therefore, was to examine the social evaluation expectancy bias and social outcome processing in individuals with SAD using a neuroscience approach.

As a brain area closely related to conflict monitoring (Botvinick et al., 2004) and pain (Eisenberger and Lieberman, 2004), the anterior cingulate cortex (ACC) has been suggested to be activated by social feedback (Somerville et al., 2006, 2010; Gunther Moor et al., 2010). For instance, a study using a Chat Room task showed that positive feedback evoked stronger activations in the ACC, as compared with negative feedback (Guyer et al., 2012). This task has also shown individual differences in ACC activity during social feedback processing (Bolling et al., 2011; Masten et al., 2011). For example, functional magnetic resonance imaging (fMRI) results have indicated that individuals with low self-esteem showed increased ACC activity in response to social rejection than those with high self-esteem (Onoda et al., 2010). One key event-related potential (ERP) component identified to be sensitive to outcome feedback is the feedback-related negativity (FRN), which is considered to be associated with the reward prediction-error mechanism located in the ACC (Holroyd and Coles, 2002; Yeung and Sanfey, 2004; Holroyd et al., 2006). This component is considered as an important biomarker in a large body of work on outcome evaluation (Gehring and Willoughby, 2002; Holroyd et al., 2004; Nieuwenhuis et al., 2004; Yeung et al., 2005; Wu and Zhou, 2009; Pedroni et al., 2011; Osinsky et al., 2014). Numerous studies have shown that the FRN is sensitive to outcome expectation (e.g., Hajcak et al., 2007), supporting the concept that the ACC is involved in predicting and signaling unexpected outcomes, regardless of their valence (Ferdinand et al., 2012). A recent study combining expectation and social feedback showed that the FRN is sensitive to both social prediction error and social rejection (Sun and Yu, 2014). Specifically, a more negative FRN waveform was observed when people were socially rejected and their explicit expectancy was violated. We thus predict that the relatively less optimistic expectancy being prevalent in individuals with SAD may lead to a larger FRN in response to positive social feedback.

Indeed, several previous ERP studies on social feedback processing have reported the effect of social anxiety on ERPs (Van der Molen et al., 2013; Kujawa et al., 2014). For example, an ERP study in young individuals using an “Island Getaway task” found a more negative FRN in response to negative than to positive feedback (Kujawa et al., 2014). More interestingly, their results also showed the influence of social anxiety on the FRN, such that a higher level of social anxiety was related to greater rejection - acceptance differentiation (ΔFRN). Besides the FRN, the significances of the P2 and P3 component for feedback processing have also been reported (Hajcak et al., 2005; Leng and Zhou, 2010; Lange et al., 2012; Schuermann et al., 2012; Flores et al., 2015). Although it remains controversial as to whether P3 can differentiate good from bad outcome, several studies have shown such a differentiation effect (Hajcak et al., 2005, 2007; Wu and Zhou, 2009). Frontal P2 is associated with the early stage of attention processing (Van der Molen et al., 2013). A number of studies have indicated that the P2 is also modulated by motivational relevance and affective significance (Cuthbert et al., 2000; Carretie et al., 2001, 2004). Moreover, a recent study reported that a strongly psychopathic group showed increased P2 and decreased P3 following reward delivering (Salim et al., 2015). In the current study, we expected to observe group differences in P2 amplitude, since individuals with higher rejection sensitivity have been reported to show a larger P2 in a modified face dot-probe task (Ehrlich et al., 2015).

To elicit the FRN in social feedback, we used the “Island Getaway task,” which is similar to the paradigm used by Kujawa et al. (2014). The primary goal of the current study was to investigate whether SAD individuals would exhibit more pessimistic expectancy in a social evaluation situation. The second aim was to examine whether the expectancy difference between a SAD and non-SAD group would manifest in the FRN amplitude during social feedback processing, particularly positive feedback. We expected that the negative expectancy in SAD would lead to a larger FRN to positive social feedback. Following the ΔFRN findings by Kujawa et al. (2014), we also expected to observe between-group differences and the influence of expectancy on ΔFRN. Specifically, we expected a larger ΔFRN for SAD group for their lower acceptance expectancy. Additionally, given that the high social rejection sensitivity was associated with larger P2 to faces (Ehrlich et al., 2015), we expected a smaller P2 for SAD group after the feedback onset due to their earlier elevated attention on faces. Considering that P3 is a classic outcome evaluation component, we also measured and analyzed this component.

## Materials and Methods

### Participants

The study was carried out in accordance with the Declaration of Helsinki and the experimental protocols were approved by the institutional review board (IRB) of Harbin Medical University. All participants provided written informed consent for the experiment.

The participants were recruited in two stages: the screen and the diagnostic interview. Two psychologists collected data in the screening stage. Additionally, two psychiatrists were in charge of the diagnostic interview in the follow-up stage of this study.

#### Screening Stage

We selected 1836 students by stratified randomized sampling; this group covered a wide range of socio-demographic status of all students attending Harbin Medical University (Da Qing Campus). These sampled students completed the Interaction Anxiousness Scale (IAS) questionnaire (Leary and Kowalski, 1993); based on a rule of thumb, after ranking the scores of the IAS, the top 27% and bottom 27% of students were classified as the high-score group and the low-score group respectively, and the remaining students were classified as the intermediate-score group (Wiersma and Jurs, 1985).

#### Follow-up Stage

All students in the high-score group were checked by the validated Chinese translation of the Structured Clinical Interview for DSM-IV (SCID-IV; Ruying et al., 1997), as the gold standard for assessing SAD; the results revealed that 102 students met the criteria for SAD. In the current study, we recruited 21 of these SAD students who agreed to participate in the electroencephalography (EEG) experiment, together with 21 matched non-socially anxious students [healthy controls (HCs)]. Due to EEG artifacts that affected data quality, the final sample consisted of 40 participants (20 SAD and 20 HC).

### Procedure

**FIGURE 1:**
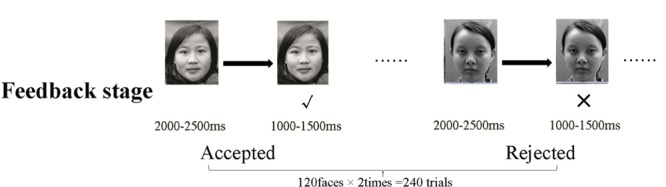
**A sample trial in the social feedback stage.** The face of the evaluator was first presented for 2000-2500 ms; then, feedback of acceptance (✓) or rejection (×) was presented below the face for 1000-1500 ms.

Three to five days before the formal experiment, participants were asked to upload their profiles with their photos, study majors, personal interests, and so on. Participants were told that they would be evaluated by another 120 peer participants (half of whom were females) based on the impression created by their profiles. To ensure the plausibility of this cover story, all participants were asked to evaluate the profiles of 10 fake participants and to vote on whether this person could remain in the group. This approach was consistent with the “Island Getaway task” in which individuals need to vote whether the presented people may remain on the island, given the limited resources (Kujawa et al., 2014). Thereafter, the formal procedure presented 60 faces of pseudo-participants with positive social feedback (i.e., social acceptance), and another 60 faces with negative social feedback (i.e., social rejection). All 120 faces were presented twice, resulting in 240 trials in total. The procedure is illustrated in **Figure 1**.

During the feedback-processing task, the face of the pseudo-participant, indicating the one who would evaluate the participant in this trial, was presented for 2000-2500 ms. Thereafter, social feedback was presented for 1000-1500 ms. An inter-trial interval was randomized from 1000 to 1500 ms, which appeared at the end of each trial (see **Figure 1**). The probability of the appearance of positive/negative social feedback was set as equal for each trial.

In the formal procedure, all instructions were presented with Microsoft PowerPoint (2013) software (Microsoft, Inc., Redmond, WA, USA). All aforementioned procedures were conducted using E-Prime software (Version 2.0, Psychology Software Tools, Inc., Pittsburgh, PA, USA).

### Expectation Rating

After the EEG procedure, participants were instructed to assess to what extent they expected their peers to accept them in real life on a scale from 0 to 100, with 0 = “Not at all” and 100 = “Very much.” Given that the number of evaluation faces used in the experiment was large (*n* = 120 in total), participants were also asked to rate how many peers they expected would accept them prior to the experiment (60 was the midpoint in this case); we also asked participants to rate their expectation after the experiment. Thus, the former rating measured real-life expectancy, while the latter measured the expectancy in the experiment.

### Electroencephalographic (EEG) Recording and Preprocessing

During the EEG recording, participants sat comfortably in an electrically shielded room approximately 80 cm from a computer screen. The EEG data was recorded using a 64-channel NeuroScan system (Neuroscan, Inc, Herndon, VA, USA). Raw EEG data were sampled at 1000 Hz/channel, referenced to the nose on-line, with impedances lower than 5 kΩ. Vertical electrooculograms (VEOGs) were recorded supra- and infra-orbitally for the left eye. Horizontal EOGs (HEOG) were recorded by electrodes at the left and right orbital rims. The online continuous data were digitized with a band-pass of 0.05-100 Hz.

Electroencephalography were re-referenced to the average of the left and right mastoids and filtered with a low pass of 20 Hz (24 dB/oct) off-line (Ferdinand et al., 2012). Epochs were feedback-locked, beginning 100 ms before feedback onset to 500 ms afterward. Ocular artifacts were removed from the EEGs using a regression procedure implemented in the Neuroscan software (Scan 4.5, NeuroScan, Inc., Herndon, VA, USA). Trials exceeding the threshold of ±80 μV were excluded from further analysis. Trials of two conditions (acceptance and rejection) were averaged, and a -100 to 0 ms baseline was used to perform a baseline correction.

### ERP Analysis

**FIGURE 2:**
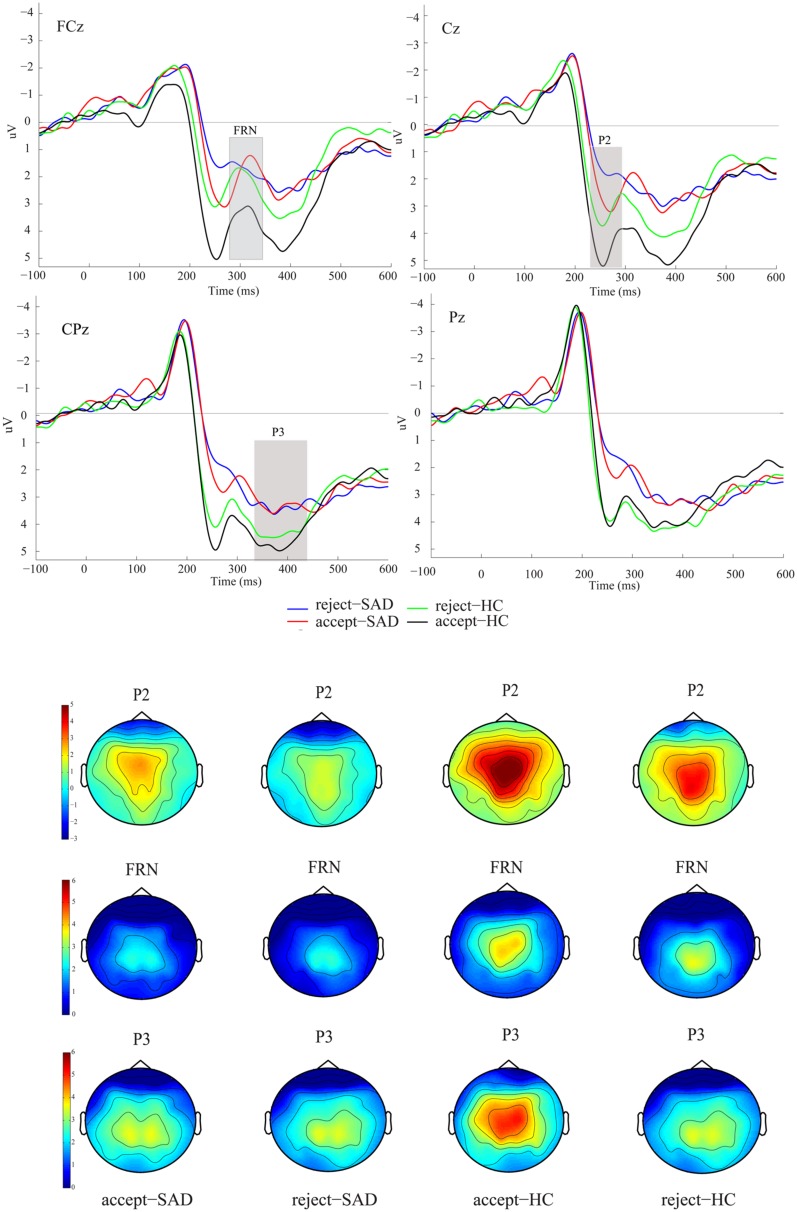
**Grand averaged event-related potentials (ERPs) and topographic maps of the two feedback types for the social anxiety disorder (SAD) and healthy control (HC) groups over midline electrodes (FCz, Cz, CPz, and Pz)**.

We were interested in the between-group difference on ERPs in both the positive and the negative feedback conditions. Therefore, we directly measured the FRN in grand-averaged waveforms rather than the difference waves between positive and negative trials. The grand-averaged ERPs at FCz and Pz and the corresponding topography map are presented in **Figure 2**. The P2 component was detected as a peak amplitude at C1, Cz, and C2 at 220-280 ms, since P2 then reached its maximum over these electrodes (see **Figure 2**). The P3 component was detected at three parietal electrodes (CP1, CPz, and CP2). The FRN was detected at three fronto-central electrodes (FC1, FCz, and FC2), which are usually used for FRN detection (Zottoli and Grose-Fifer, 2012; Luo et al., 2014). Visual observation of the topography map supported the above selections (see **Figure 2**). The original FRN amplitude was measured for each participant as the peak amplitude within the 280–340 ms window. However, considering that the P2 was also sensitive to negative vs. positive difference and group difference in our study, we used a peak-to-peak measurement here to eliminate the potential influence of P2 on the FRN (Ferdinand et al., 2012). Therefore, the reported FRN results are based on the difference of the FRN and P2 amplitudes. The P3 was identified as the average amplitude within the 340-450 ms window. To directly compare with the finding of Kujawa et al. (2014), we also analyzed the negative minus the positive amplitude difference (ΔFRN), in which more negative values reflect heightened reactivity to negative vs. positive feedback. The averaged P2, P3, and FRN amplitudes were entered into a 2 (feedback valence: positive vs. negative) × 2 (group: SAD vs. HC) analysis of variance (ANOVA). In addition, the ΔFRN was incorporated into a two-sample *t*-test with group as the between-subject variable. The reported degrees of freedom of the F-ratio were corrected by using the Greenhouse–Geisser method when the sphericity assumption was violated.

## Results

### Patient Demographics

The basic information of the participants is provided in **Table 1**. An independent-samples *t*-test revealed that the two groups differed significantly in anxiety scores, but not in age or gender ratio. All participants had normal vision (with correction), and were right-handed.

**Table 1:** Characteristics and self-reported measures of participant groups.

	SAD group (*n* = 20)	Healthy controls (*n* = 20)	*t*-test (*df* = 38)
Age in years *(SD)*	20 (1.11)	20.42 (0.74)	1.476
Gender (% females)	63.6%	61.9%	χ^2^ test, *p* = 0.74
IAS *(SD)*	51.05 (11.36)	30.29 (5.60)	-7.337^∗∗∗^
STAI			
Trait anxiety *(SD)*	49.64 (13.75)	33.67 (7.60)	-4.317^∗∗∗^
State anxiety *(SD)*	47.68 (14.44)	30.38 (7.36)	-4.539^∗∗∗^
SES *(SD)*	24.41 (6.31)	32.71 (2.68)	5.229^∗∗∗^

*SAD, social anxiety disorder; IAS, Interaction Anxiousness Scale; SES, self-esteem scale; STAI, Chinese version of Spielberger’s trait anxiety inventory (STAI); ^∗∗∗^p < 0.001.*

### Feedback Expectancy Results

The expectancy probabilities of peer acceptance in both real life and in the experiment were analyzed. The two sample *t*-test showed that the SAD participants (*M* = 58.5%, *SD* = 13.96) showed significantly lower peer-acceptance expectancy in real life than did the HCs (*M* = 78.95%, *SD* = 15.09; *t*_38_ = 4.614, *p* < 0.001). Similarly, SAD participants (*M* = 43.5%, *SD* = 13.89) also had significantly more negative-acceptance expectancy in the experiment than did HC participants (*M* = 58.62%, *SD* = 11.13; *t*_38_ = 3.847, *p* < 0.001).

The two types of expectancy probability were positively correlated (*r* = 0.492, *p* < 0.001). Moreover, the IAS score was negatively correlated with both social acceptance expectancy in real life (*r* = -0.663, *p* < 0.001) and acceptance expectancy in the experiment (*r* = -0.421, *p* < 0.01).

### ERP Results

**Figure 2** shows the ERPs elicited by the two types of feedback at the midline electrodes (FCz, Cz, CPz, and Pz).

### The P2 Component

Analysis of variance on P2 amplitudes revealed a significant main effect of feedback valence (*F*_1,38_ = 16.09, *p* < 0.001, $\eta_{p}^{2}$ = 0.297), such that positive social feedback (*M* = 4.60 μV, *SE* = 0.43) evoked a larger P2 than did negative social feedback (*M* = 3.53 μV, *SE* = 0.41). Furthermore, the P2 amplitude also indicated a significant main effect of group (*F*_1,38_ = 8.63, *p* < 0.01, $\eta_{p}^{2}$ = 0.185), such that the SAD group (*M* = 2.91 μV, *SE* = 0.56) had a smaller P2 than did the HC group regardless of feedback valence (*M* = 5.23 μV, *SE* = 0.56).

### The FRN

For the peak-peak FRN amplitudes, ANOVA indicated a significant main effect of feedback valence (*F*_1,38_ = 7.84, *p* < 0.01, $\eta_{p}^{2}$ = 0.171), showing that the FRN of positive social feedback (*M* = -3.14 μV, *SE* = 0.33) was larger than that of negative social feedback (*M* = -2.41 μV, *SE* = 0.31). Furthermore, the feedback valence × group interaction effect (*F*_1,38_ = 5.79, *p* < 0.05, $\eta_{p}^{2}$ = 0.132) indicated that only SAD participants showed such a positive vs. negative FRN effect (positive: *M* = -3.40 μV, *SE* = 0.47, negative: *M* = -2.04 μV, *SE* = 0.43), whereas HC participants did not (positive: *M* = -2.90 μV, *SE* = 0.45, negative: *M* = -2.77 μV, *SE* = 0.46, *p* = 0.72).

Furthermore, the ΔFRN (rejection – acceptance) analysis showed that SAD participants had a more positive ΔFRN (*M* = 1.36 μV, *SD* = 1.60) than did HCs (*M* = 0.10 μV, *SD* = 1.69; *t*_38_ = -2.41, *p* < 0.05).

### The P3 Component

Analysis of variance on P3 failed to find any social feedback effect or group-related effect (*F*s < 1.30, *p*s > 0.27).

### Correlations between Behavioral and ERP Results

A bivariate correlation analysis showed that the peer-acceptance expectancy in real life correlated negatively with the ΔFRN (*r* = -0.469, *p* < 0.01). Interestingly, the IAS score correlated positively with the ΔFRN (*r* = 0.342, *p* < 0.05). No other significant correlation was detected.

### Mediation Analysis Results

**FIGURE 3:**
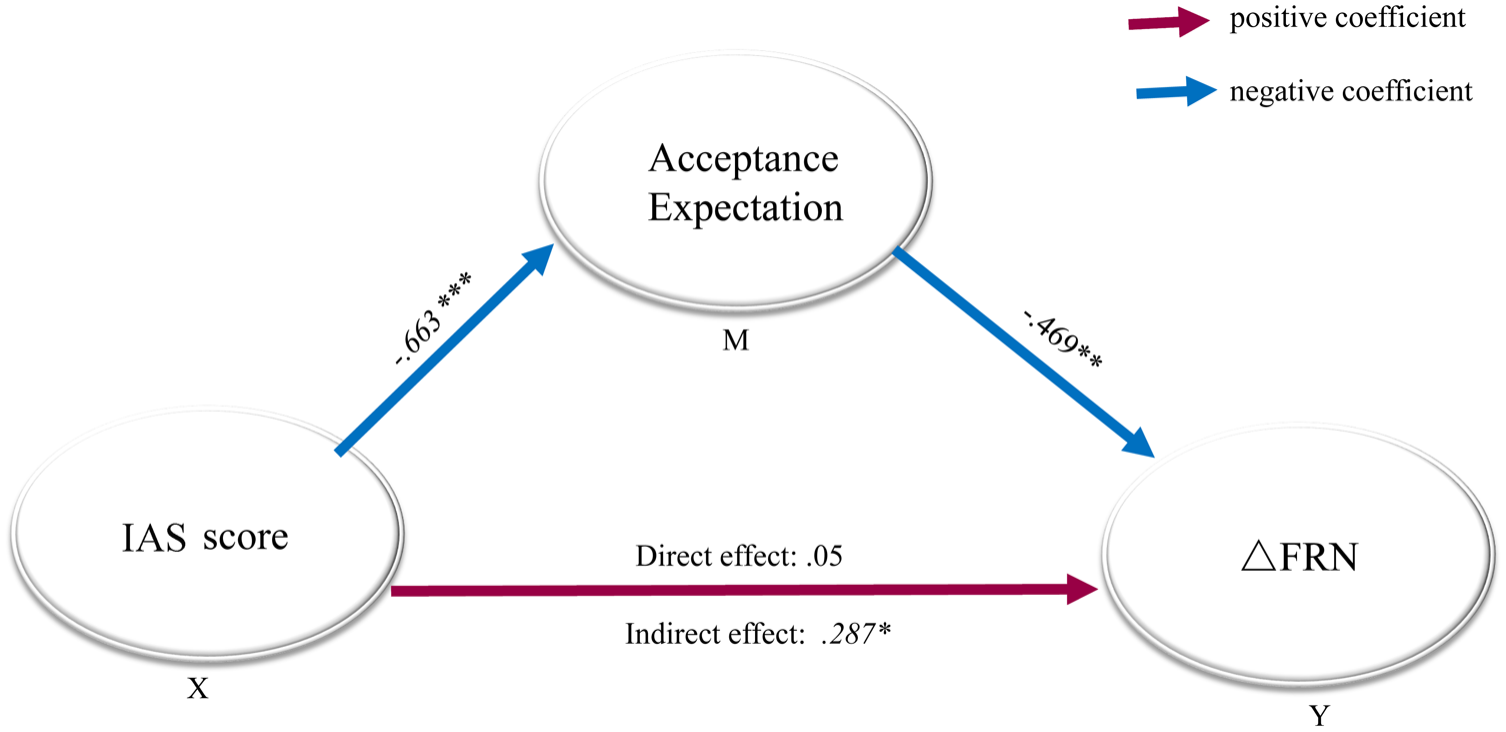
**Mediation model with standardized regression coefficients for the relationship between social anxiety and ΔFRN.** Mediation model with standardized regression coefficients showing the relationship between social anxiety and ΔFRN as mediated by acceptance expectancy. The standardized regression coefficient between the Interaction Anxiousness Scale (IAS) score and ΔFRN, controlling for acceptance expectancy, was 0.05. Social anxiety (IAS score) predicted the acceptance expectancy (B = -0.663), which in turn predicted ΔFRN (B = -0.469). The direct effect of IAS on ΔFRN (when expectancy bias was included in the model) was not significant, *p* = 0.77, while the indirect effect was significant, indicating that acceptance expectancy fully mediated the relationship between IAS and ΔFRN. X: predictor, Y: outcome variable, M: mediate variable, ^∗^*p* < 0.05; ^∗∗^*p < 0.01*; ^∗∗∗^*p* < 0.001.

We conducted a mediation analysis to assess whether the acceptance expectancy in real life lays in the causal path between the IAS score and the FRN amplitude, using a bootstrapping number of 5000 (Preacher and Hayes, 2008). In the analysis model, ΔFRN was set as the outcome variable, acceptation expectancy in real life served as the mediator, and the IAS score was entered as the predictor (see **Figure 3**), and the analysis was performed as described by (Preacher and Hayes, 2004). First, we found that the direct effect in the model with acceptance expectancy was not significant (B = 0.56, *SE* = 0.19, *p* > 0.77). Second, a significant indirect effect of social anxiety through acceptance expectancy was confirmed (B = 0.287, *SE* = 1.34, *p* = 0.025) at a 95% bias-corrected confidence interval (95%, CI: 0.0105-0.5624), establishing that acceptance expectancy was in the causal path between social anxiety and ΔFRN.

## Discussion

The primary aim of the present study was to investigate whether people with SAD exhibit less positive expectancy in social situations than healthy people, and to what extent this kind of expectancy bias manifests in the ERPs. By using a social feedback task, we found that SAD participants had less positive social acceptance expectancy in both real life and experimental social situations than did HCs. The ERP findings of FRN showed a more negative FRN in response to positive feedback than to negative feedback in SAD individuals. We interpret this finding as indicating that lower expectation is associated with a larger FRN. Thus, this result reflects the cognitive negative bias in SAD. Moreover, the correlation between acceptance expectancy, IAS and ΔFRN amplitude further confirms that highly socially anxious participants showed a larger positive vs. negative differentiation, since this was mediated by acceptance expectancy.

### Larger FRN for Positive Social Feedback vs. Negative Feedback in SAD

Previous studies on anxious participants have reported individual differences in the FRN, such that larger FRN amplitudes are associated with lower levels of anxiety (Gu et al., 2010a,b; Simons, 2010; Takács et al., 2015). In the current study, the SAD group showed more negative FRN values for positive than for negative social feedback. We suggest that this result may indicate the negative expectancy in SAD participants. Indeed, behavioral measures showed that their expectancy rate of being accepted was 43.5% in the experiment situation, which was significantly lower than the random level (50%, *p* < 0.05). Such an inference effect was consistent with the prediction of the response-outcome theory (Alexander and Brown, 2010, 2011) that FRN is related to subjective expectancy, regardless of feedback valence.

An alternative interpretation of the larger FRN to positive feedback in SAD individuals is the blunting of the FRN after negative feedback in SAD (see **Figure 2**). That is, considering that the FRN is also related to performance monitoring (see review Ullsperger et al., 2014), a dysfunction in the social performance monitoring processes of anxious participants may result in weaker sensitivity to negative feedback. In parallel with this hypothesis, previous studies in depression (Foti and Hajcak, 2009) have also shown a blunted FRN on non-reward feedback. A recent study on problematic internet use (PIU; Yau et al., 2015) also indicated overall decreased sensitivity to feedback in individuals with PIU, which manifested as a reduced FRN. For the purpose of social adaptation, negative feedback is of great significance for adjusting social behavior to be more favorable (Ruff and Fehr, 2014). Individuals with SAD may have impaired negative social feedback processing, which is reflected by blunted neural responses to negative feedback.

The absence of differences in FRN between negative and positive feedback in HCs is consistent with some previous studies that did not report an FRN differentiation effect (Bolling et al., 2011; Leitner et al., 2014; Dekkers et al., 2015). For example, with a similar social feedback paradigm, Leitner et al. (2014) did not find any feedback effect on the FRN. However, the absent positive vs. negative feedback effect seems inconsistent with previous studies which showed that the FRN is sensitive to the valence of social feedback, and is more negative in response to rejection than to being accepted (Kujawa et al., 2014; Sun and Yu, 2014). We posit that the paradigm used, or the ERP measurement itself, may contribute to such inconsistent findings. Regarding the paradigm, in Sun and Yu’s (2014) study, Somerville’s task was used, which presented the expectation and feedback simultaneously, was used, while only the feedback from the peer was presented in our task. Regarding the measurement, the FRN effect is strongly dependent on how the FRN is quantified. In Kujawa et al.’s (2014) work, the FRN was scored as the original FRN mean amplitude, but not as the peak-to-peak amplitude. In contrast, we reported peak-to-peak amplitude results due to the potential P2 influence. When using the same measurement in the original research of Kujawa et al. (2014), the main effect of feedback, i.e., that negative feedback evoked more negative FRN, was also observed.^1^ The current study was unable to determine whether either or both of the above factors contribute to the absence of positive vs. negative effect. Further research is required to clarify this issue.

### Relationship between Expectation, Social Anxiety, and ΔFRN

ΔFRN is an index of the level of differentiation between negative and positive feedback, which was found to be sensitive to individual differences in social anxiety in a previous study (Kujawa et al., 2014). In the current study, we further established the link between expectancy, social anxiety and ΔFRN. First, SAD participants showed a larger ΔFRN (negative – positive) than did HCs. Moreover, this index was correlated with acceptance expectancy in real life as well as with the IAS score. More specifically, individuals with high acceptance expectancy in real life exhibited a smaller ΔFRN, and individuals with high social anxiety exhibited a larger ΔFRN in response to social feedback. Finally, mediation analysis confirmed that the acceptance expectancy in real life fully mediated the correlation between social anxiety and ΔFRN.

In line with the cognitive-behavior model of social anxiety (Rapee and Heimberg, 1997), it is conceivable that social expectancy mediated the FRN effect in social feedback processing. That is, an increase in social interaction anxiety predicted a decline in social acceptance expectancy in real life, which in turn predicted the FRN difference between the response to social positive vs. negative feedback. For SAD individuals, negative beliefs about social situations lead to their negative expectancy of future social evaluation (Caouette et al., 2015). Such negative expectancy also influences social evaluation processing, which is reflected by a more negative FRN to positive social feedback and a more negative ΔFRN (negative – positive). The dysfunction in social evaluation differentiation (indicated by a larger ΔFRN) may further reinforce SAD individuals’ cognitive symptoms or negative beliefs during social life. In line with the existing studies that proposed the FRN as a biomarker in psychopathology (Olvet and Hajcak, 2008; Proudfit, 2015), we suggest that the ΔFRN in response to social feedback may serve as a potential biomarker of SAD.

### The Social Feedback Valence Effect and Group Effect on P2

Although there have been many ERP studies on social rejection, few studies have reported the P2 effect (Sreekrishnan et al., 2014). Our results showed a smaller P2 for social rejection, which is consistent with a previous study on autism spectrum disorder subjects that also showed a smaller P2 for rejection, regardless of the group difference (McPartland et al., 2011).

In the current study, there was a between-group difference in P2: the HC group showed a larger P2 to social feedback than did the SAD group. A smaller P2 in anxious participants than in non-anxious participants has also been observed in a study using fear stimuli (Frenkel and Bar-Haim, 2011), in which non-anxious participants showed overall larger ERPs (P1, P2, early posterior negativity). The anxiety-related attenuation of early P2 was also found in social distance processing (Perry et al., 2013). Given these findings, we consider that the reduced P2 associated with social rejection in the SAD group reflects reduced engagement of attentional resources during the early stage of social feedback processing (McPartland et al., 2011). Such a smaller P2 for SAD individuals may reflect a critical social avoidance; i.e., smaller P2 amplitudes in SAD indicates an early avoidance response after the face of the evaluator was presented, since socially anxious individuals tend to avoid social contact (Amir et al., 1998; Heuer et al., 2007; Lange et al., 2008; Heitmann et al., 2014).

## Conclusion

To sum up, both groups showed an positive vs. negative differentiation effect on the P2, which also showed a between-group difference. This result might reflect a shared early social evaluation sensitivity mechanism for both socially anxious and non-anxious individuals, although it might also sensitive to the level of social anxiety. Furthermore, SAD participants exhibited a larger FRN to positive social feedback and a blunted FRN to negative social feedback, demonstrating their dysfunction in feedback processing. Combining the ERP findings, and the correlation and the mediation effect for ΔFRN, our results indicated that ΔFRN is a potential biomarker for SAD.

## Conflict of Interest Statement

The authors declare that the research was conducted in the absence of any commercial or financial relationships that could be construed as a potential conflict of interest.

## Footnotes

1 When we analyzed the original FRN mean amplitude, the ANOVA indicated a significant main effect of feedback valence (*F*_1,38_ = 5.737, *p* < 0.05, $\eta_{p}^{2}$ = 0.131), indicating more negative FRN in response to negative feedback.
